# Fourth‐generation glucose sensors composed of copper nanostructures for diabetes management: A critical review

**DOI:** 10.1002/btm2.10248

**Published:** 2021-09-09

**Authors:** Gowhar A. Naikoo, Tasbiha Awan, Hiba Salim, Fareeha Arshad, Israr U. Hassan, Mona Zamani Pedram, Waqar Ahmed, Hakkim L. Faruck, Alaa A. A. Aljabali, Vijay Mishra, Ángel Serrano‐Aroca, Rohit Goyal, Poonam Negi, Martin Birkett, Mohamed M. Nasef, Nitin B. Charbe, Hamid A. Bakshi, Murtaza M. Tambuwala

**Affiliations:** ^1^ Department of Mathematics and Sciences College of Arts and Applied Sciences, Dhofar University Salalah Oman; ^2^ Department of Biochemistry Aligarh Muslim University Aligarh India; ^3^ College of Engineering Dhofar University Salalah Oman; ^4^ Faculty of Mechanical Engineering—Energy Division K.N. Toosi University of Technology Tehran Iran; ^5^ School of Mathematics and Physics College of Science, University of Lincoln Lincoln UK; ^6^ The Hormel Institute University of Minnesota Austin Minnesota USA; ^7^ Departmnt of Pharmaceutics and Pharmaceutical Technology Yarmouk University Irbid Jordan; ^8^ School of Pharmaceutical Sciences Lovely Professional University Phagwara Punjab India; ^9^ Biomaterials and Bioengineering Lab Translational Research Centre San Alberto Magno, Catholic University of Valencia San Vicente Mártir Valencia Spain; ^10^ School of Pharmaceutical Sciences Shoolini University of Biotechnology and Management Sciences Solan India; ^11^ Department of Mechanical and Construction Engineering Northumbria University Newcastle upon Tyne UK; ^12^ Department of Pharmacy School of Applied Science, University of Huddersfield UK; ^13^ Department of Pharmaceutical Sciences Rangel College of Pharmacy, Texas A&M University Kingsville Texas USA; ^14^ School of Pharmacy and Pharmaceutical Science Ulster University Coleraine UK

**Keywords:** diabetes management, early detection, electrode materials, hybrid copper nanostructures, nonenzymatic glucose sensors

## Abstract

More than five decades have been invested in understanding glucose biosensors. Yet, this immensely versatile field has continued to gain attention from the scientific world to better understand and diagnose diabetes. However, such extensive work done to improve glucose sensing devices has still not yielded desirable results. Drawbacks like the necessity of the invasive finger‐pricking step and the lack of optimization of diagnostic interventions still need to be considered to improve the testing process of diabetic patients. To upgrade the glucose‐sensing devices and reduce the number of intermediary steps during glucose measurement, fourth‐generation glucose sensors (FGGS) have been introduced. These sensors, made using robust electrocatalytic copper nanostructures, improve diagnostic efficiency and cost‐effectiveness. This review aims to present the essential scientific progress in copper nanostructure‐based FGGS in the past 10 years (2010 to present). After a short introduction, we presented the working principles of these sensors. We then highlighted the importance of copper nanostructures as advanced electrode materials to develop reliable real‐time FGGS. Finally, we cover the advantages, shortcomings, and prospects for developing highly sensitive, stable, and specific FGGS.

## 1 INTRODUCTION

Glucose is the primary source of energy in living cells and plays a critical role in biology. Diabetes can result in elevated blood glucose levels that pose a severe hazard to human health.^1^, ^2^ Diabetes is an overgrowing global public disease and is characterized by insufficient insulin formation or distribution in the body, causing the death of 1.6 million people per year worldwide.^3^, ^4^, ^5^, ^6^ It is a chronic condition that requires daily monitoring of blood glucose levels,^7^ and in severe cases, insufficient insulin levels can result in diabetic ketoacidosis, leading to seizures.^8^ Diabetes complications can also include neuro and cardiovascular diseases in addition to kidney disorders^9^ and new risks such as heart failure, kidney dysfunction, poor vision, nerve damage, and disability.^10^, ^11^, ^12^ These complications often result due to poor blood glucose control. Regulated and routine blood glucose tests are necessary when coping with emergencies, including hypoglycemia (low blood sugar level).^13^, ^14^, ^15^ Detecting glucose levels rapidly and reliably in clinical and biological samples remains a major challenge.^16^, ^17^ Limiting sugar consumption and continuously tracking blood glucose levels is critical to managing diabetes and can significantly reduce life‐threatening diabetes and provide sufferers with a healthy lifestyle.^18^, ^19^, ^20^, ^21^, ^22^ Electrochemical glucose sensors^23^, ^24^, ^25^ with high sensitivity, good selectivity, rapid test, low‐cost, reliable, and accurate in situ detection,^26^, ^27^, ^28^, ^29^ have attracted great attention when compared to other sensing technologies like chemiluminescence,^30^ surface‐enhanced Raman scattering,^31^ mass spectrometry,^32^ calorimetry,^33^ fluorescence spectroscopy,^34^, ^35^ and optical sensors.^36^ In addition, substantial efforts have been made to investigate glucose sensing in various potential fields such as the pharmaceutical industry, pathology, physiology, food processing, and bio‐fermentation.^37^ Glucose sensors account for approximately 85% of the biosensors industry because they represent the direct health consequences of diabetes, which affects over 400 million people worldwide.^38^, ^39^, ^40^ Reliability and economic glucose sensors with good sensitivity and low detection limits are crucial to combat the prevailing situation. This current review targets the rapid development and recent advances of Cu‐based electrochemical biosensors for glucose detection. It summarizes all the generations of electrochemical glucose sensors, followed by the fundamentals of fourth‐generation glucose sensors (FGGS). In the end, future challenges and perspectives for further development of Cu‐based FGGS are proposed.

Electrochemical sensors are used to monitor blood glucose levels rapidly.^41^ These devices allow for real‐time detection. Furthermore, continuous glucose monitors have been used to enable autonomous insulin delivery, where glucose measurements automatically adjust insulin delivery in closed‐loop systems. In this manner, insulin can be administered to the patient in cases of hyperglycemia.^41^ Enzymatic glucose sensors (EGS) are based on glucose oxidase or glucose dehydrogenase enzymes and exhibit a very high and reliable sensitivity.^42^ However, some limitation of such sensors, including chemical and thermal conditions, instability, and relatively high complexity of the test samples.^43^ Fluctuations in external factors like pH, humidity level, and temperature, and so on, hinder further exploration in the field of enzyme‐based glucose biosensors.^44^, ^45^, ^46^

**FIGURE 1:**
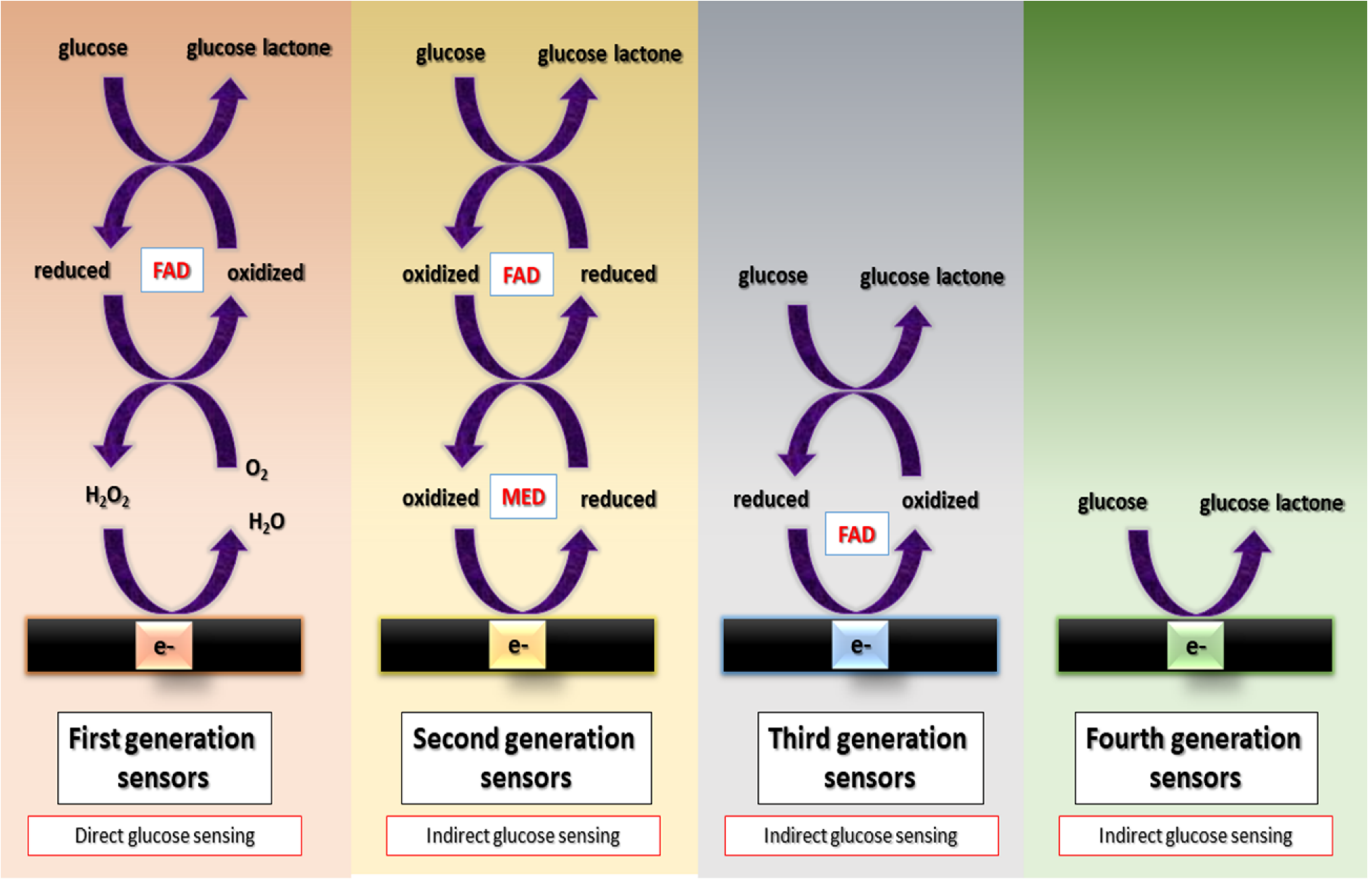
A description of the mechanisms of enzymatic glucose oxidation in first‐, second‐, third‐, and fourth‐generation glucose sensors^3^

Enzymes‐based glucose sensors are divided into three significant generations.^47^, ^48^, ^49^ The first generation requires free oxygen to immobilize the enzyme (GOx) on the electrode. Oxygen dependency of these sensors has limited applications in oxygen‐deficient blood samples.^15^, ^50^ The second generation of enzyme‐based glucose sensors included an artificial mediator, which directly reacts with the enzyme glucose oxidase leading to less sensitivity and accuracy. Artificial mediators involved one‐electron reversible redox ferrocene derivatives and ferrocyanide.^51^ The third generation was investigated to compensate for the shortcomings of the previous generations. However, minor changes in pH, temperature, and humidity were still susceptible to enzymatic denaturation.^3^, ^12^ The immobilization of enzymes on the conducting electrode's surface is complex, and its quantity cannot be precisely controlled. The high cost, complicated fabrication procedure, short shelf life, and poor reproducibility of enzyme‐based glucose sensors have always been challenging for researchers.^52^, ^53^ A description of enzymatic glucose oxidation mechanisms, viewed as first‐, second‐, and third‐generation sensors, is depicted in Figure 1.^3^ The aforementioned disadvantages of EGS attracted researchers to develop fourth‐generation metal‐based enzyme‐free glucose sensors (FGGS)^54^, ^55^, ^56^ that oxidize glucose directly on the electrode surface.^57^, ^58^, ^59^ FGGS that do not rely on enzymes have gained widespread attention^60^ and are considered ideal for glucose analysis because of their low cost, efficient sensitivity, high selectivity, and good stability.

## 2 WORKING PRINCIPLE OF FGGS

Among the electrochemical detection techniques, two basic methods, amperometry and potentiometry, have been widely used.^15^, ^50^ The potential difference between a reference electrode and a working electrode is determined in potentiometric sensors at zero applied currents. The potential of the working electrode varies with the concentration of glucose. It has been shown that these sensors can evaluate glucose concentrations of 10 M or higher (an average human's blood glucose level is in the range of 4–7 mM).^12^ Nonenzymatic electrodes have recently been developed by combining various metals and metal nanoparticles (NPs), including metal/metal oxide and alloy composites, for high sensitivity and low detection limit of the FGGS.^64^ Bimetallic NPs can also be used in FGGS due to their superior electronic properties and increased catalytic activity. Similarly, alloys and metal oxides can be employed because they improve glucose oxidation and reduce the poisoning in the sensing electrodes of the sensor.^43^

The previous decade has seen extensive advancements in the working mechanisms and principles of FGGS.^61^, ^62^, ^63^ Like metal oxide‐based non‐EGS (NEGS), the copper‐based FGGS functions at varying pH. The functioning of the sensor depends on the stimulation of the metal oxide surface. This occurs in the vicinity of highly reactive hydroxide ions, which also serve the catalytic purpose during the oxidation of glucose molecules. Tian et al. developed the following mechanism for glucose sensing using copper oxide‐based NEGS.^64^

$$\mathrm{CuO}+{\mathrm{OH}}^{-}\to \mathrm{Cu}{\left(\mathrm{OH}\right)}_{2}+e^{-},$$

$$\mathrm{Cu}{\left(\mathrm{OH}\right)}_{2}+{\mathrm{OH}}^{-}\to \text{CuOOH}+H_{2}O+e^{-},$$

$$\text{CuOOH}+C_{6}H_{12}O_{6}\;\left(\text{glucose}\right)\to \mathrm{Cu}{\left(\mathrm{OH}\right)}_{2}+C_{6}H_{10}O_{6}\;\left(\text{gluconolactone}\right).$$

**FIGURE 2:**
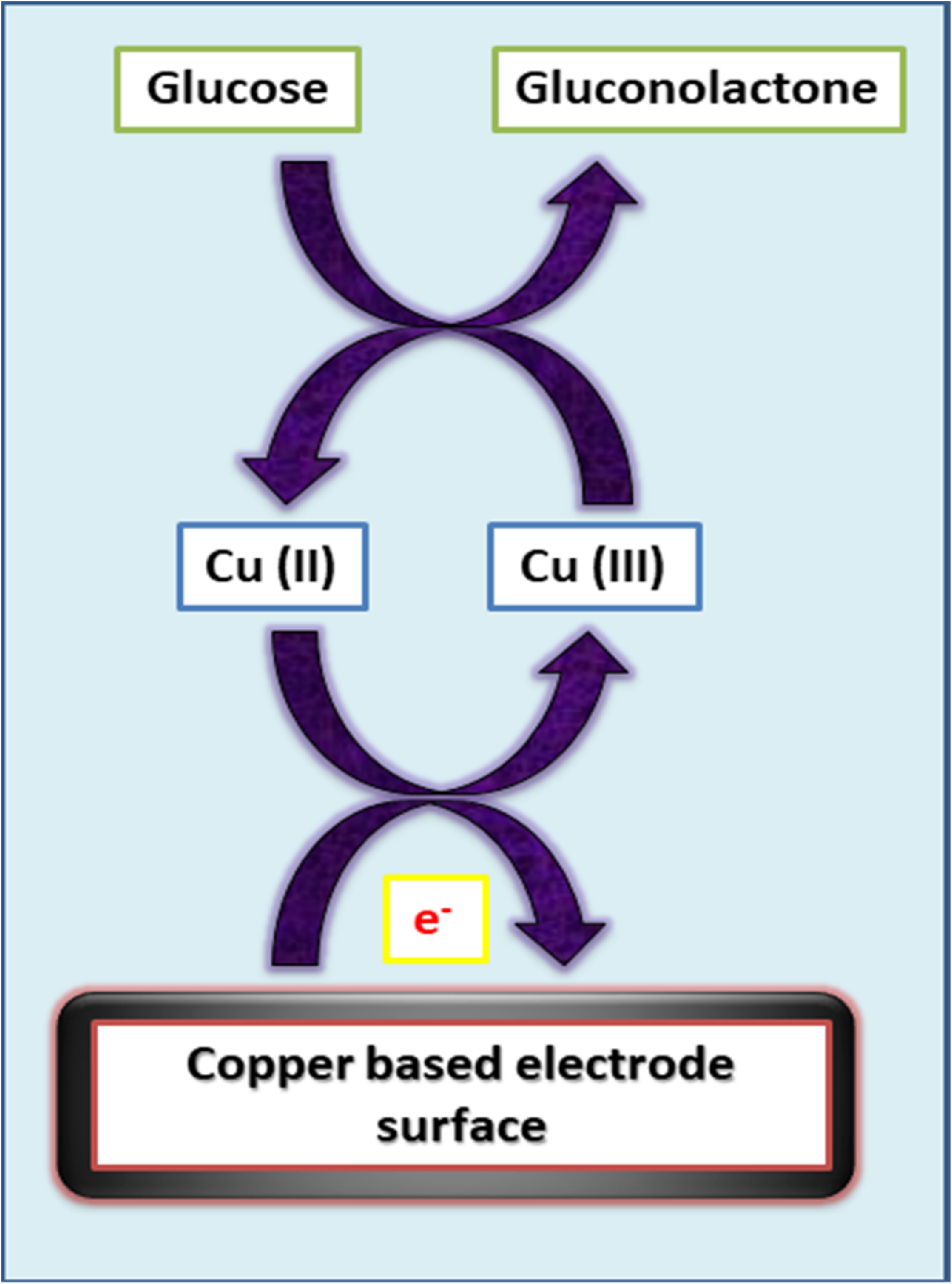
Reactions that occur in a copper‐based fourth‐generation glucose sensors (FGGS)

The mechanism of this reaction is based on the electrochemical function of copper oxide that changes its oxidation states during the reaction.^65^, ^66^ This is evident in the above‐mentioned chemical reactions that occur in the FGGS. During the sensing process, as the voltage shifts, Cu^2+^ cations present in CuO get oxidized to Cu^3+,^ and CuOOH is formed (Figure 2). This then allows the oxidation of glucose to develop gluconolactone in the next step of the reaction. During the same stage, Cu^3+^ gets reduced to Cu^2+^, leading to the formation of CuO or Cu(OH)_2._ ^64^, ^67^ These step‐by‐step reactions cause a shift in the transfer rate of electrons on the electrode surface, thereby causing an increase in the overall electrical current generated. This is then recorded by the sensing detector, which detects glucose molecules in the given sample.

**FIGURE 3:**
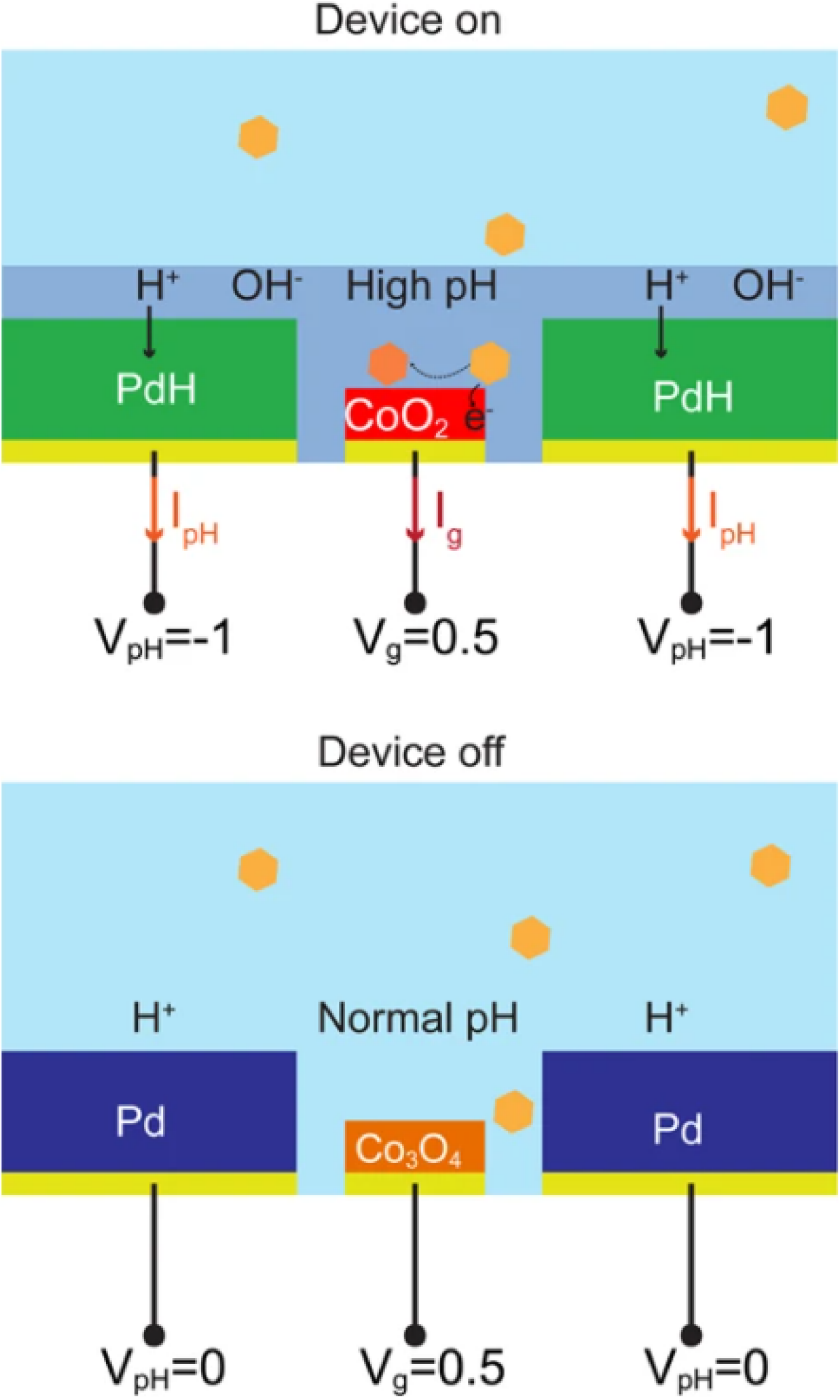
Operating principle for glucose sensing. When the device is on (top), *V*_pH_ = −1 V, the Pd contact absorbs H^+^ from the solution and increases its pH. At high pH, the Au/Co_3_O_4_ contact is in its more reactive CoO_2_ oxidized state. With *V*_g_ = 0.5 V, the CoO_2_ contact oxidizes glucose and the resulting *I*_g_ is collected, which increases with increased glucose concentration. When the device is off (bottom), *V*_pH_ = 0 V, the pH is at physiological values, typically pH 7, no sensing occurs from the Au/Co_3_O_4_ and *I*_g_ = 0 A. Reprinted with permission from Reference 134, Copyright @ 2019 (Nature)

Metal oxide‐based NEGS like Co_3_O_4_, MnO_2_, CuO, Cu_2_O have almost similar glucose‐sensing mechanisms (Figure 3). This could occur by any or all of the following three methods^62^: (1) the copper oxide gets activated under strongly alkaline conditions, (2) formation of intermediary by‐products that function as a catalyst to oxidize glucose molecules, and (3) the intermediary by‐products then undergo reduction to give the original copper oxide.

### 2.1 Mechanism of FGGS

The CuO‐based glucose sensors combined with gold NPs (AuNPs) and modified with CuO nanowires electrode (CuO NWs) gave a linear range of 0.5 μM to 5.9 mM and sensitivity of 4398.8 μA mM^−1^ cm^−2^ and a rapid response rate of 5 s.^68^ The synergistic mechanism proposed for CuO in alkaline media requires oxides, hydroxides, and oxyhydroxides for the electrochemical oxidation of glucose.^69^ The strong catalytic properties of Cu and its derivatives have been reported to accelerate glucose oxidation.^110^ Cu in CuO is electrochemically oxidized to strong oxidizing species such as Cu(OH)^−4^ or CuOOH^−^. Thus, the +2 oxidation state changes to +3^111^:

$$\mathrm{Cu}+{\text{2OH}}^{-}\to \mathrm{CuO}+H_{2}O+2{e^{-}}_{,}$$

$$\mathrm{CuO}+{\mathrm{OH}}^{-}\to \text{CuOOH}+e^{-},\text{OR},$$

$$\mathrm{CuO}+H_{2}O\to {{\text{CuOH}}_{4}}^{-}+{e^{-}}_{.}$$

**FIGURE 4:**
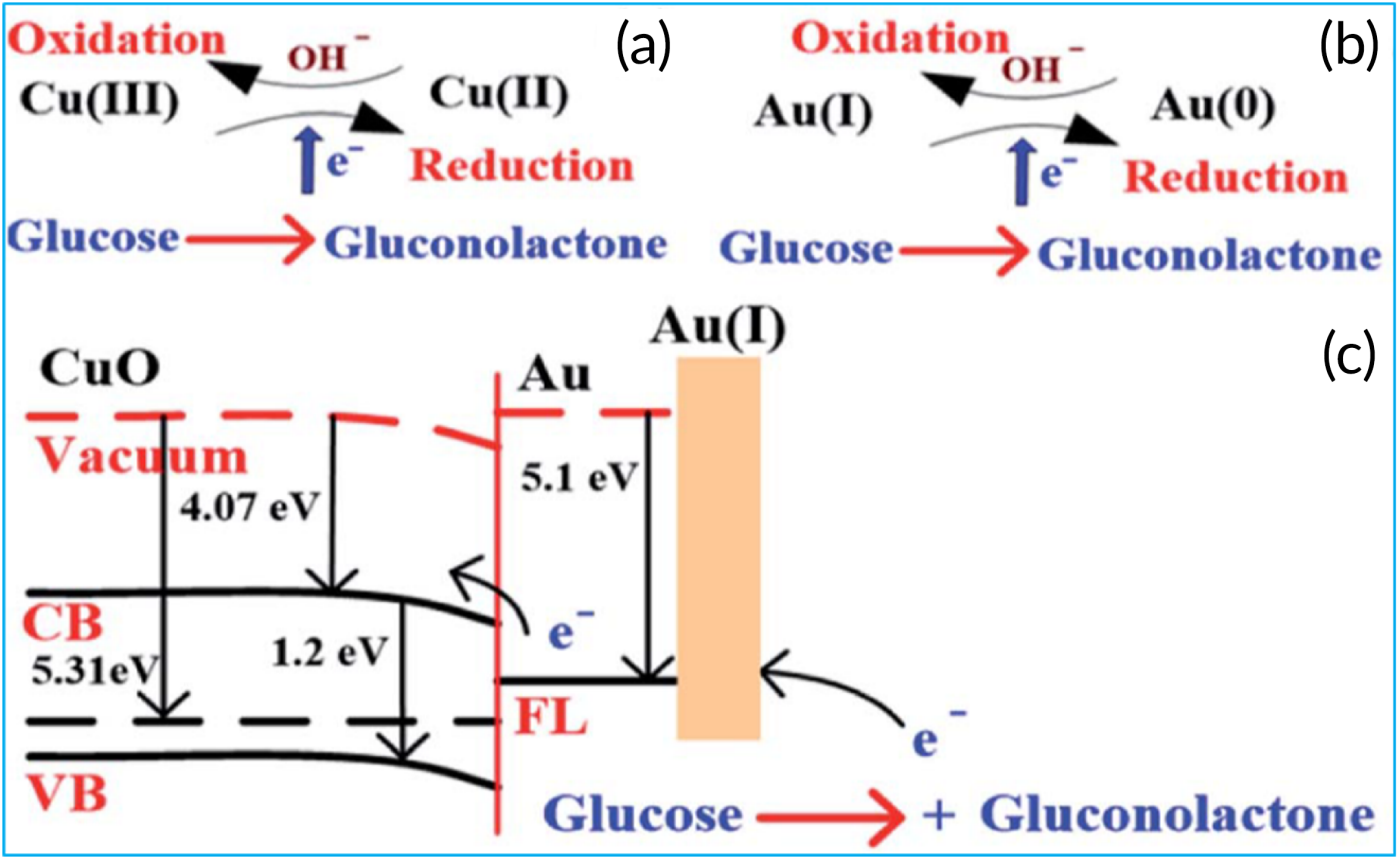
(a) Glucose detection mechanism in CuO, (b) glucose detection mechanism in Au, and (c) glucose detection mechanism in CuO nanowires (CuO NWs)/Au nanoparticle (AuNP) structure under applied potential. Reproduced with permission from Reference 68, Copyright @ 2019 (The Royal Society of Chemistry)

Cu(III) catalyzes glucose's oxidation into gluconolactone and hydrolyzed into gluconic acid, as shown in Figure 4.^112^ The reduction of Cu(III) to Cu(II) can be demonstrated by oxidation and reduction peaks. Cu(III) is the most responsible medium for electron transfer compared to other valence Cu ions. The stability of the AuNPs modified CuO NWs electrode was also investigated for more than 10 days with an interval of 2 days, which showed comparatively better stability than a bare CuO NWs electrode. The high catalytic capability of CuO NWs/AuNPs compared to bare CuO NWs could be attributed to incorporating AuNPs on the surface of CuO NWs, which significantly enhances the surface volume ratio of the designed electrode. The reported glucose sensor's properties were highly effective and reliable in testing human blood. Because of its high sensitivity and low limit of detection (LOD), it is suitable for noninvasive glucose detection in saliva and urine.

$$\mathrm{Cu}\left(\mathrm{III}\right)+\text{Glucose}+e^{-}\to \text{Gluconolactone}+\mathrm{Cu}\left(\mathrm{II}\right),$$

Gluconolactone→Gluconic Acid.

The sensitivity, detection range, detection limit, and response time of FGGS composed of copper and copper oxide nanostructures are given in Table 1.

**TABLE 1:** Comparison of the performance of different Cu‐based nonenzymatic glucose sensors

Electrodes/samples/electrocatalysts	Sensitivity (μA mM^−1^ cm^−2^)	Applied potential (V)	Limit of detection (LOD) (μM)	Linear range (mM)	Response time (s)	References
Green synthesis of Cu spherical NPs	1065.21	—	0.046	1–7.2	<3	119
CuO PN	3072	0.6 V Ag/AgCl	0.41	0.005–0.225 and 0.225–0.825	∼0.8	120
CuO‐flower	2062	0.5 V Ag/AgCl	0.25	0.001–1	1.6	121
Cu_2_O/Cu/CC	6952	0.60 V Hg/HgO	0.06	0.001–1.555	<2	124
Cu‐MOF/MWNTs/GCE)	3878	—	0.4	0.0005–11.84	0.3	126
Au NPs‐modified CuO NWs	4398.8	0.6 V Ag/AgCl	0.5	0.0005–5.9	Approx. 5	68
CuO‐ZnO NRs/FTO	2961.8	—	0.40	0.001–8.45	<2	122
Cu_*x*_O nanosheets/Cu	1541	0.60 V Ag/Agl	0.57	4	∼3	152
Cu_3_(BTC)_2_‐derived CuO nanorod	1523.5	0.6 V Ag/AgCl	1	Up to 1.25	5	153
Cu/Ni/Au nanoporous film	4135 2972	—	0.1	0.0005–3 3–7	—	154
Cu^+2^/PANI/rGO/FR4 nanocomposite	4168.37 525.4	0.66 V Ag/AgCl	4.93	0.0028–0.0222 0–4	<5	155
CuS nanosheets/Cu_2_O/CuO NWAs/Cu foil	4262	0.60 V Ag/AgCl	0.89	0.002–4.1	350–800	156
Copper oxide/CPE	1183.59	—	672.8	1.6–62.5	120	157
MWCNT‐CuBTC	14,949	0.6	10	0.2–1	—	158
MOF‐derived CuO architectures	10–120	—	0.1	0.01–0.12	~6	159
CuO/CuBi_2_O_4_	330	—	0.7	0.000001–100	—	160
CuS microflowers	1007	0.5	—	0.2–5.4	~4	161
Cu2O‐c/SPCE	2376.7	−1.0 to 1.2 V. Ag/AgCl	0.003	0.000031–1.42	—	162
Cu^2+^/MWCNT‐COOH	1732	—	0.02	0.00002–8.0	~2	163
CuO NPs/PEDOT:PSS/PGE	663.2	+0.70 V	—	10	—	164
CuO hollow sphere	25.0 ± 0.8	—	—	0.001–3	—	165
CuO hollow sphere	13.6 ± 0.3	—	—	3–11.5	—	165
CuO microspheres	26.59	—	20.6	2–9	—	166
Cu‐GNE	—	0.5	0.12	1	~2	167
Cu NWs/PANI/rGO	843.06	0.64	1600	0–4	—	168
CuO‐C‐dots	110 and 63.3	+0.50	200	0.5–2 and 2–5	—	169
Cu_*x*_O/Cu	1210 ± 124	−0.2 and +0.6	10	0.01–7	~1	133
CuO nanoleaves	1467.32	+0.6	0.012	0.005–5.89	~3.5	104
CuNPs/PoPD/GCE	—	0.5	0.25	0.005–1.6	~1	170
CoNiCu alloy nanotubes	791	—	0.5	0.05–1.551	—	106

Abbreviations: BTC, benzene tricarboxylate; CC, carbon cloth; CuBTC, copper‐1,3,5‐benzenetricarboxylic acid; Cu‐GNE, Cu nanoparticles on a linear graphene edge nanoelectrode; Cu‐MOF, Cu‐metal–organic frameworks; CPE, carbon paste electrode; FTO, fluorine doped tin oxide; GCE, glassy carbon electrode; MWCNT, multiwall carbon nanotubes; MWNTs, multiwalled carbon nanotubes; NPs, nanoparticles; NRs, nanorods; NWAs, nanowire arrays; PANI, polyaniline; PN, porous nanostructure; rGO, reduced graphene oxide.

## 3 COPPER NANOSTRUCTURES AS ADVANCED ELECTRODE MATERIALS FOR FGGS

A variety of nanostructured electrocatalysts are being investigated to develop advanced FGGS, as simple electrodes cannot compete with their level of glucose detection.^65^, ^66^, ^67^ FGGS, which are based on the electrochemical oxidation of glucose that operates through a variety of inorganic catalysts including noble metals (Ag, Au, Pt, and Pd) and their alloys (Pt–Pd, Pt–Au, and Au–Pd), metal oxides (Co_3_O_4_, NiO, CuO, Cu_2_O, ZnO, and so on) or bimetallic electrodes and carbon‐based nanomaterials have been extensively explored for their excellent^68^, ^69^ glucose detecting capabilities.^70^, ^71^, ^72^ Pt‐based nanosensors have shown high catalytic efficiency owing to their large surface area and their ability to control the kinetics of the reaction.^76^ However, Pt and its derivatives are costly, which limits its practical application.^77^ The same has been observed with Au‐ and Pd‐based NEGS. Therefore, widespread applications of noble metals have been hampered by disadvantages like low selectivity, high cost, toxicity, and metal scarcity, making their use impractical on a larger scale production.^73^, ^74^

In addition, metallic,^75^, ^78^, ^79^, ^80^, ^81^ metal‐alloy based,^82^, ^83^, ^84^ metal hydrate,^85^ metal sulfide,^86^ and metal oxides^87^, ^88^, ^89^, ^90^ have been successfully used to study nonenzymatic glucose sensing. These metallic‐based glucose sensors make use of a variety of sensing techniques like the fluorescent carbon dots‐based fluorescent method,^91^ optical methods,^92^ Raman spectroscopy,^93^ and others. To achieve efficient reproducibility of glucose sensors, the continuous development of nanomaterials from other transition metals and their oxides is widely researched.^94^ Subsequently, researchers are excited about FGGS since they can compare different transition metals with excellent redox activity and select those with superior stability and selectivity.^95^, ^96^ Transition metal oxides and their alloys, such as ZnO, CuO, NiO, and CO_3_O_4,_ are widely used for glucose biosensors due to their high electrochemical activity, low cost, and the low potential requirement for electron transfer reactions.^97^, ^98^, ^99^, ^100^

**FIGURE 5:**
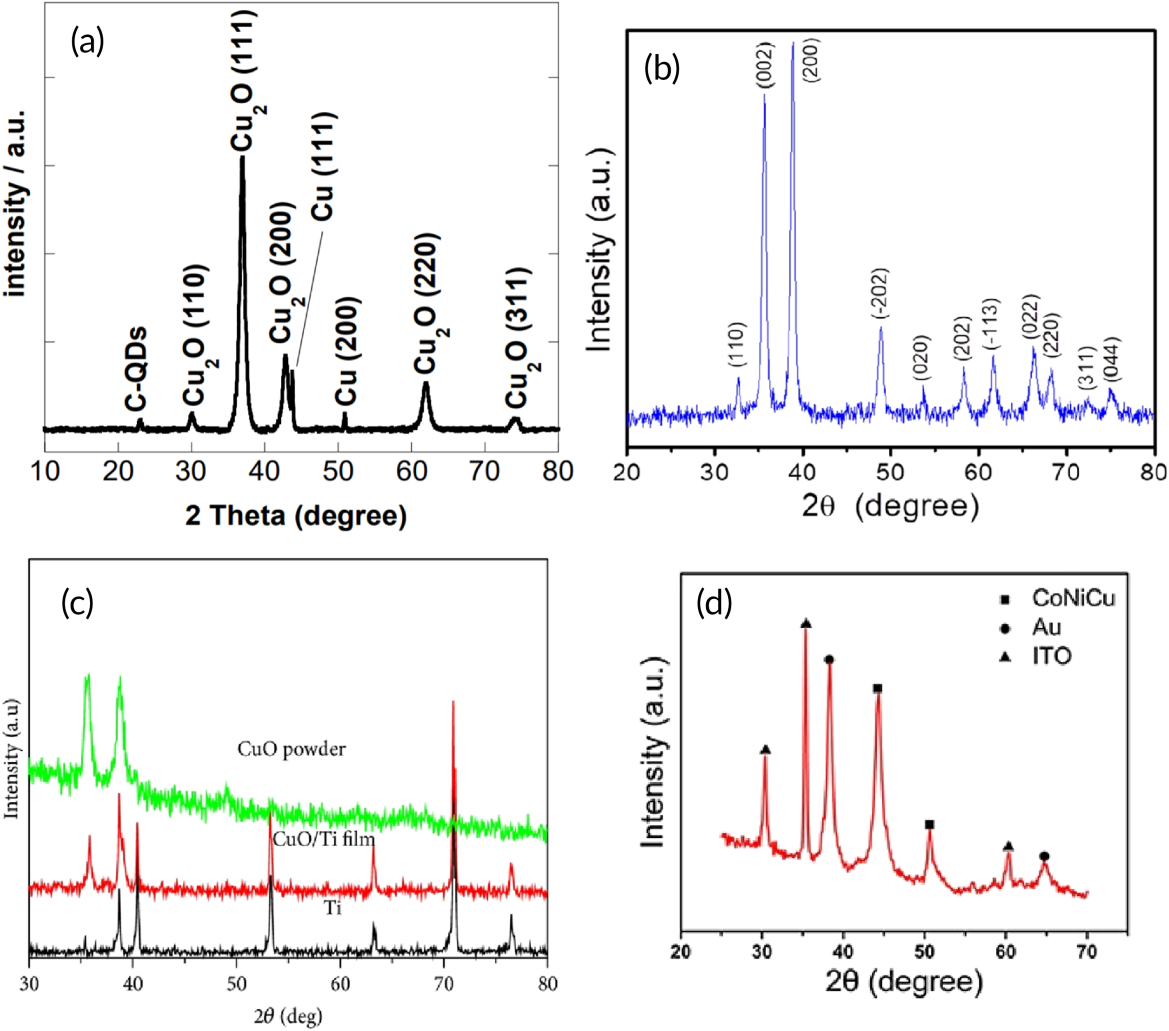
X‐ray diffraction (XRD) characterization of copper particles used in nonenzymatic glucose (NEGS). (a) XRD pattern of carbon quantum dots loaded with copper oxide nanoparticles (CQDs/Cu_2_O NPs). Adapted with permission from Reference 103, Copyright @ 2016 (MDPI). (b) XRD pattern of CuO nanoleaves. Adapted with permission from Reference 104, Copyright @ 2021 (IOP Science). (c) XRD patterns of Ti substrate, CuO film on Ti substrate, and CuO powder. Adapted with permission from Reference 105, Copyright @ 2014 (Hindawi). (d) XRD patterns of prepared CoNiCu alloy nanotubes arrays transferred on indium tin oxide. Adapted with permission from Reference 106, Copyright @ 2019 (Frontiers)

Copper oxides (CuO and Cu_2_O) are particularly significant among transition metal oxides because of their excellent thermal, mechanical, and chemical stability. Much attention has been paid to developing copper oxide electrode materials for FGGS with high electrocatalytic activities.^101^, ^102^ X‐ray diffraction (XRD) characterization of copper particles, their oxides, carbon quantum dots loaded with copper oxide NPs (CQDs/Cu_2_O NPs), or CoNiCu alloy nanotubes (NTs) arrays transferred on indium tin oxide, helps to determine their crystal structure, orientation, shape, and size as well as other structural parameters such as average grain size, strain and crystal defects, characteristics that are important for FGGS applications^103^, ^104^, ^105^, ^106^ (Figure 5).

### 3.1 Electrochemical detection of glucose using copper‐based FGGS

Because of their outstanding chemical and thermal stability, various nanomaterials exhibit remarkable sensitivity and selectivity in glucose sensing.^82^ Their electrochemical properties, high electrode catalytic activity, low cost, strength, natural abundance, nontoxicity, and environmentally friendly nature^107^, ^108^, ^109^, ^110^ have made Cu and its oxides a potential candidate for various applications such as photoelectric devices, gas sensing devices, lithium‐ion batteries, and especially as electrochemical sensors due to their optical nature and electrical characteristics.^82^, ^83^, ^84^ CuNPs are an effective electrode material for glucose detection and are extremely sensitive to glucose oxidation due to their excellent electrical conductivity.^85^ CuNPs have a high specific surface area, which improves FGGS activity significantly, and their synthesis techniques have evolved to include hydrothermal, pyrolysis, and electrodeposition.^113^, ^114^, ^115^, ^116^, ^117^ CuO is a p‐type semiconductor with a narrow bandgap of 1.2 eV, which is more stable than simple Cu for glucose analysis. CuO nanomaterials show excellent electrocatalytic activity, proper redox potential, and low overpotential during electron transfer experiments.^91^, ^92^, ^93^ CuO and CuS act as excellent electronic mediators in glucose oxidation due to the redox pairs of Cu^2+^ and Cu^3+.^ ^94^

**FIGURE 6:**
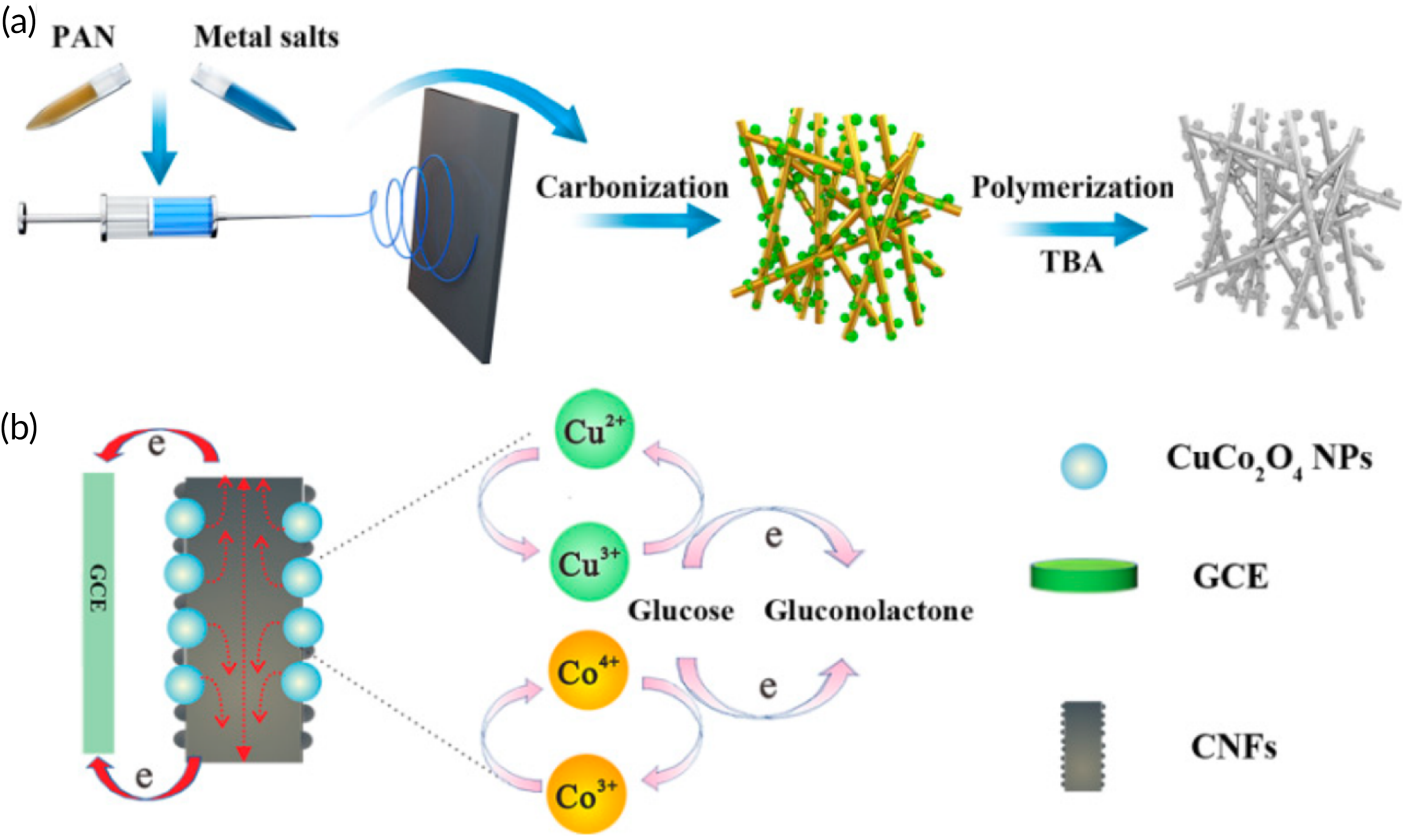
(a) Schematic diagram for the preparation of CuCo_2_O_4_–carbon nanofibers (CNFs) and (b) the representation of the proposed mechanism for electrocatalytic oxidation of glucose based on poly(thiophene‐3‐boronic acid) (PTBA)/CuCo_2_O_4_–CNFs/glassy carbon electrode (GCE). Reproduced with permission from Reference 118, Copyright @ 2019 (MDPI)

Various CuO nanostructures have been extensively researched and synthesized into multiple shapes with individual properties and performance through the development of nanotechnology, such as NPs, nanorods, nanofibers, nanospheres, flower‐like structures, and so on.^95^, ^96^ In a study by Ding et al., they developed an FGGS based on CuCo_2_O_4_ using electrospinning technology and carbonization treatment to prepare CuCo_2_O_4_–carbon nanofibers (CNFs)^118^ (Figure 6). This sensor exhibited an enhanced activity with two linear ranges of 0.01–0.5 and 0.5–1.5 mM and a high sensitivity of 2932 and 708 μA mM^−1^ cm^−2^.

A simple, cost‐effective microwave‐based method for synthesizing a sensitive FGGS with CuO nanodisks was investigated, which maintained its remarkable selectivity, a high sensitivity of 627.3 μA·mM^−1^·cm^−2^, and broad linear range from 2.0 M to 2.5 mM.^98^ Furthermore, glucose sensors showed high reproducibility and longstanding stability with only 9% sensitivity damage in 14 days with an interval of 2 days in the open air. The sensing ability of the proposed electrode was evaluated in human urine samples and can be attributed to the development of noninvasive biosensors in ambient conditions. The shape and size of CuO nanodisks composed of tiny nanorods are confirmed by transmission electron microscopy (TEM) while their high crystalline nature is determined from selected area electron diffraction patterns.

Dayakar et al. fabricated FGGS with pristine CuNPs on glassy carbon electrode (GCE). They prepared this sensor by the simple green method using leaf extract of *Ocimum tenuiflorum*.^99^, ^119^ Less toxic, smooth surface, and small‐sized NPs synthesized via this green method outperformed the catalytic activity toward glucose oxidation than other synthesized nanostructures. Furthermore, the modified Cu/GCE electrode exhibited the current response, which remained at 93.2% of its original value when stored for 10 days at room temperature, reflecting its long‐term stability toward glucose oxidation. The proposed electrode presented outstanding analytical sensing properties such as reproducibility, limited interference, and a sensitivity of 1065.21 μA mM^−1^ cm^−2^, with a detection limit of 0.038 μM (S/N = 3), and linear response ranges from 1 to 7.2 mM with a fast response of 3 s.

CuO porous nanostructure (CuO PN) electrodes have been shown to enhance glucose detection capabilities, showing a linear range of 0.005–0.225 mM, a high sensitivity of about 3072 μA mM^−1^ cm^−2^, and a low detection limit of about 0.41 μM.^100^ Furthermore, the proposed sensor maintained its high stability over a month of monitoring, recording a 17% loss in current density under regular measurements. Thus, this cost‐effective and highly stable porous CuO FGGS was used for detecting glucose in human saliva with a high sensitivity of ∼2299 μA mM^−1^ cm^−2^.

**FIGURE 7:**
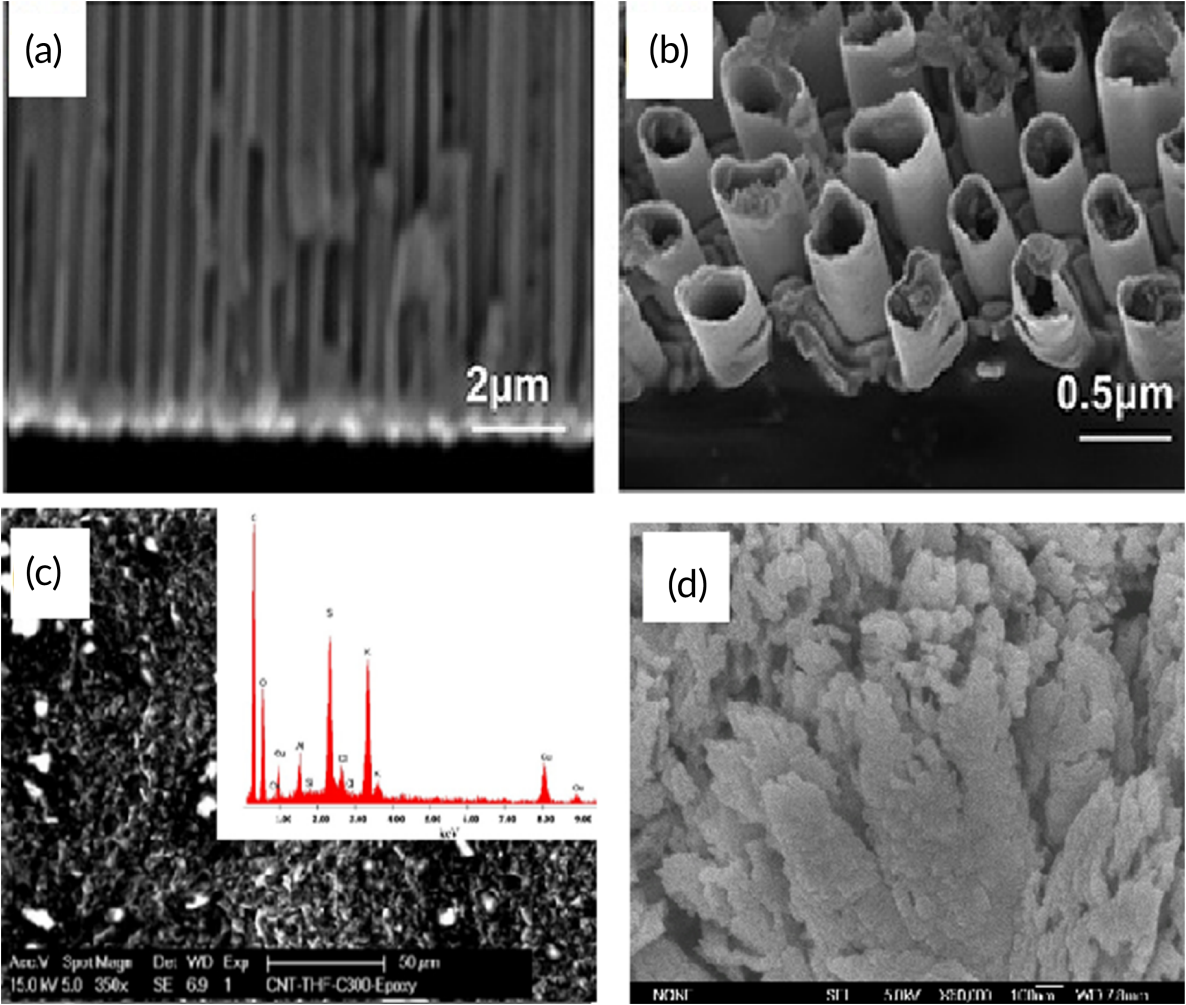
Scanning electron microscopy (SEM) characterization of copper particles used in NEGS. (a,b) Products deposited on CoNiCu alloy nanotubes with anodic aluminum oxide template. Adapted with permission from Reference 106, Copyright @ 2019 (Frontiers). (c) MWCNT‐copper‐1,3,5‐benzentricarboxylic acid (CuBTC) composite electrode. Adapted with permission from Reference 158, Copyright @ 2020 (MDPI). (d) CuO film on Ti substrate. Adapted with permission from Reference 105, Copyright 2014 (Hindawi)

Ashok and colleagues synthesized CuNPs using three methods, the colloidal method with NaBH_4_ as a reducing agent producing the best homogeneous phase of CuO NPs (Cu‐colloids).^121^ Simultaneously, combustion‐based techniques, which were exploring glycine (Cu‐gly) and hydrazine (Cu‐hyd), did not yield any satisfactory results.^101^, ^102^ Flower‐shaped Cu‐colloidal particles showed a maximum electro‐oxidation current of glucose, with a low detection limit of 0.25 μM, a high sensitivity of 2062 μAmM^−1^ cm^−2^ for glucose, in a wide linear range of 1–850 μM. The Cu‐colloid particles' excellent electrocatalytic activity is related to their unique blossomed flower‐shaped morphology and the pointed tips of opened mesoporous or microporous petals.^121^ This structure provided more active sites with a large surface area, promoting chemisorption of oxygen and charge transportation. The scanning electron microscopy (SEM) analysis demonstrates the morphology and composition of the Cu particles (Figure 7).

Most Cu or Ni‐based NEGS are synthesized by modifying the substrate with NPs, scattered structures, or metal‐carbon hybrids.^103^ In addition to CuO, nanostructures of cuprous oxide (Cu_2_O) have also been investigated to study their electrocatalytic properties to fabricate FGGS. Zhang et al. synthesized a self‐supported Cu_2_O/Cu/CC (carbon cloth) using a single step, simple potentiostatic electrochemical deposition on CC.^104^, ^105^ The flexible glucose sensor (Cu_2_O/Cu/CC) demonstrated a superior sensitivity of 6952 μA mM^−1^ cm^−2^, reproducibility (relative standard difference [RSD] = 2.74%) with an extremely low detection limit of 0.6 μM with a fast response time of less than 2 s. Moreover, 90.2% of the original sensitivity of the electrode was maintained during a 1‐month stability test.

**SCHEME 1:**
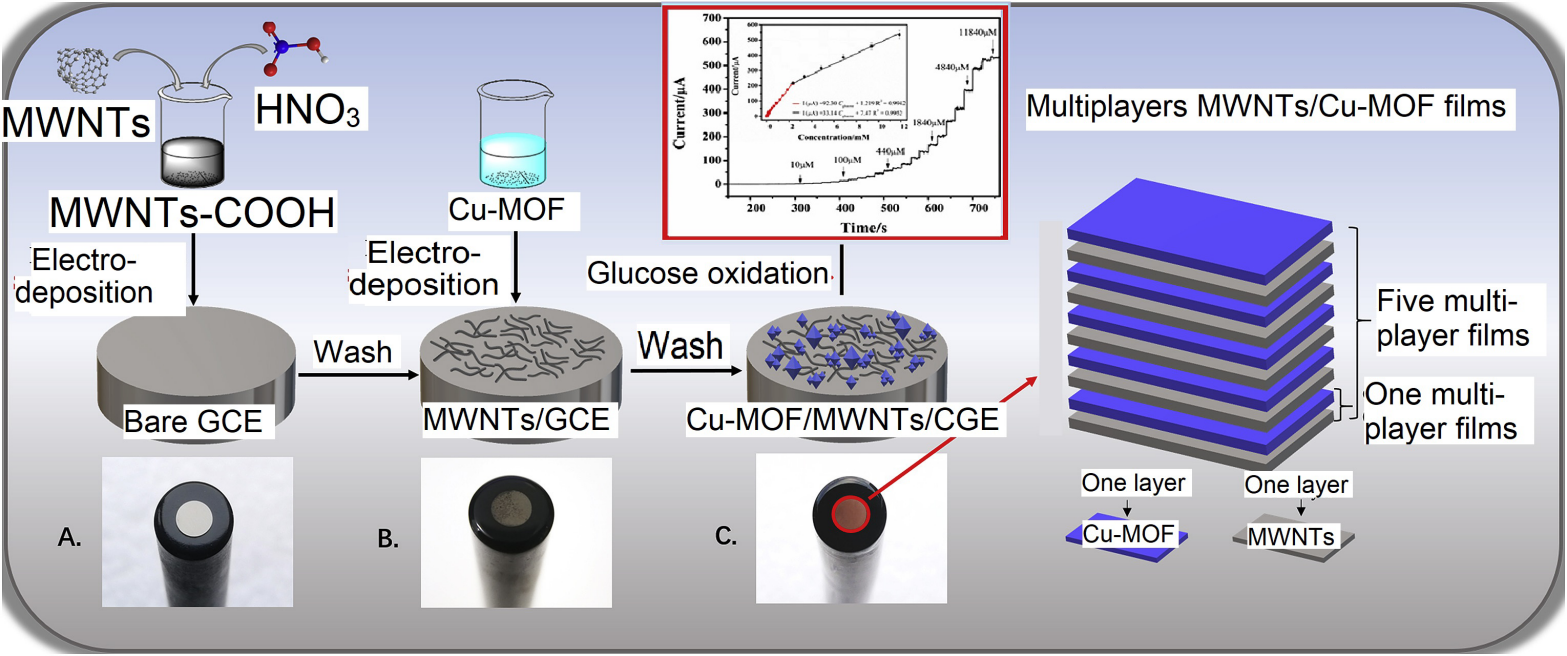
Schematic illustration of the Cu‐metal organic frameworks (Cu‐MOF) and multilayer films of Cu‐MOF/multiwalled carbon nanotubes (MWNTs)/glassy carbon electrode (GCE). Reproduced from Reference 126, Copyright 2019 (Elsevier)

The sensitivity and conductivity of the purest Cu electrode can be easily contaminated by oxidation; thus, researchers are focused on improving their efficiency by incorporating other components.^125^ Wu et al. manufactured a high‐performance multilayer composite film‐based FGGS through a layer‐by‐layer method, employing Cu‐metal organic frameworks (Cu‐MOF), multiwalled carbon NTs (MWNTs) modified GCE, given in Scheme 1.^107^, ^126^ The glucose sensor showed an excellent sensitivity of 3878 μA mM^−1^ cm^−2^, a more comprehensive linear range of 0.5 μM–11.84 mM, with a low LOD of 0.4 μM and was free of interference. Researchers found that the hybrid composite's enhanced catalytic properties were due to its large surface area, multiple active sites, accounting for its excellent electrical conductivity of MWNTs and good selectivity of Cu‐MOF. The glucose detection efficiency of the developed sensor was tested in actual blood samples, yielding satisfactory and feasible results. However, due to the high alkaline conditions, its practical application was hindered.

### 3.2 Advantages and challenges of copper‐based FGGS

FGGS have received widespread attention in recent times because of their ability to deliver reproducible results with higher stability than traditional EGS. This, therefore, increases the practicability of FGGS in clinical applications and several recent studies support this advantage.^57^, ^123^, ^127^, ^128^, ^129^ Furthermore, these NEGS remain functional even after 1 month and monitor glucose levels in undiluted whole blood after sterilization and thus exhibit long‐term stability.^24^, ^130^, ^131^ This was previously not feasible with traditional glucose monitors that are active only for 7–14 days and get inactivated due to biofouling. In addition, NEGS show low detection time and rapid response rate, which adds to the advantage of these sensors over EGS. This was seen in the work by Yan et al., who demonstrated the practical clinical application of an FGGS based on copper sulfide nanoflakes‐reduced graphene oxide that showed a rapid response rate as low as 6 s and a low detection limit of 0.19 μM in human blood and urine samples.^132^

Hence, recently the scientific focus has shifted toward developing nanomaterials‐based NEGS that provide better linear range and ease in operation. Nanomaterials also possess sizes equivalent to enzyme molecules that aid in their functionalization. Highly conductive carbon‐based nanomaterials are the best choice for electro‐oxidation of glucose; however, their stability is a significant concern. As a result, researchers have concluded that copper and its bimetallic nanomaterials have a promising potential for fostering and promoting FGGS in mass production. Because of its unusual electrocatalytic activity and use in many electrochemical devices, copper‐based FGGS have gained widespread popularity in recent decades. Copper is abundantly available in nature, low cost, and environmentally friendly and shows high catalytic activity.^113^, ^114^ Moreover, copper‐based FGGS with other metals in combination have displayed excellent sensing properties. In research carried out by Suneesh and colleagues, an FGGS based on Co–Cu alloy NPs was developed, which served as an excellent sensing device for quantifying glucose levels.^113^

Despite the innumerable research done on FGGS and the use of nanomaterials in their construction, there are still a few challenges that need to be tackled before these sensors can be availed. The major obstacles that need to be addressed include miniaturization of the sensor, reduction in the sample required during sensing, and quick delivery of results. Furthermore, more work is necessary to improve the shelf life and reduce the cost of such test strips based on NEGS. The application of nanomaterials in the development of FGGS has also gained widespread attention, especially to create sweat based platforms and in vivo implantable glucose sensors for glucose measurements. However, such sensors to detect glucose concentrations in these fluids would require greater sensitivity of copper‐based FGGS. Also, these sensors show a highly accurate correlation between glucose levels measured in interstitial fluids and blood when measured using the commercially available blood‐glucose meter. This correlation has been observed in several recent studies,^134^, ^135^, ^136^, ^137^, ^138^ and thus, such sensors can be potentially used in clinical applications for glucose measurements.

In addition, the biocompatibility and shelf life of copper‐based FGGS depend on the morphology of the copper architectures and the attached functional groups.^138^ This is especially true in the case of implantable sensors made using NPs. Immune reaction against NPs is inevitable; hence, such reactions can be controlled by monitoring the size and shape of the NPs that indirectly influence the attachment of neutrophils or macrophages to them.^139^, ^140^, ^141^ For example, particles that are not spherical and are over 6 μm in diameter may exhibit lowered macrophage adhesions and, therefore, will have better functional viability within the system.^138^ Hence, the physical features of the NPs, their chemical nature, like their surface chemistry, influence their biocompatibility and enhance the sensor's overall life.^139^

Furthermore, there is no sophisticated control over the protective sheath, thickness, and pore size of the nanoporous layer that would allow FGGS to work on plasma, human serum, and blood when undiluted. Moreover, disturbances caused by various electro‐active and electro‐inactive chemical species must still be adjusted.^61^ Therefore, although these sensors offer promising alternatives to traditional, invasive blood glucose monitoring; further works need to be done produce better electrode protective films in FGGS, before these sensors are made available commercially on a large scale.

### 3.3 Comparison of FGGS composed of Cu nanostructures with EGS

The current glucose‐sensing devices available are based on EGS. A few studies have also demonstrated the catalytic effect of copper oxide in enzymatic glucose oxidation and hydrogen peroxide detection with good stability and ultra‐sensitive response. Umar et al., for example, established a reproducible glucose biosensor based on well‐crystallized flower‐shaped CuO nanostructures formed of thin nanosheets.^142^ The designed biosensor showed a response time of less than 5 s, a high sensitivity of 47.19 μA mM^−1^ cm^−2^, and a LOD of 1.37 M. Several studies have shown that combining graphene with CuO NPs will produce more synergistic results and hence improve glucose detection. For instance, Qian et al. suggested a simple and straightforward method for depositing Cu_2_O NPs on graphene sheets (Cu_2_O@CRG) using sodium citrate as a reluctant agent,^143^ demonstrating better sensitivity and selectivity in alkaline media than Cu_2_O or CRG. However, these sensors possess several drawbacks related to the inherent nature of the enzymes, like their minimal reproducibility and decreased stability when used for long durations. Also, the catalytic function of enzymes is easily affected by the external pH, temperature, absence or presence of humid conditions, and other chemicals in the vicinity.^144^

**FIGURE 8:**
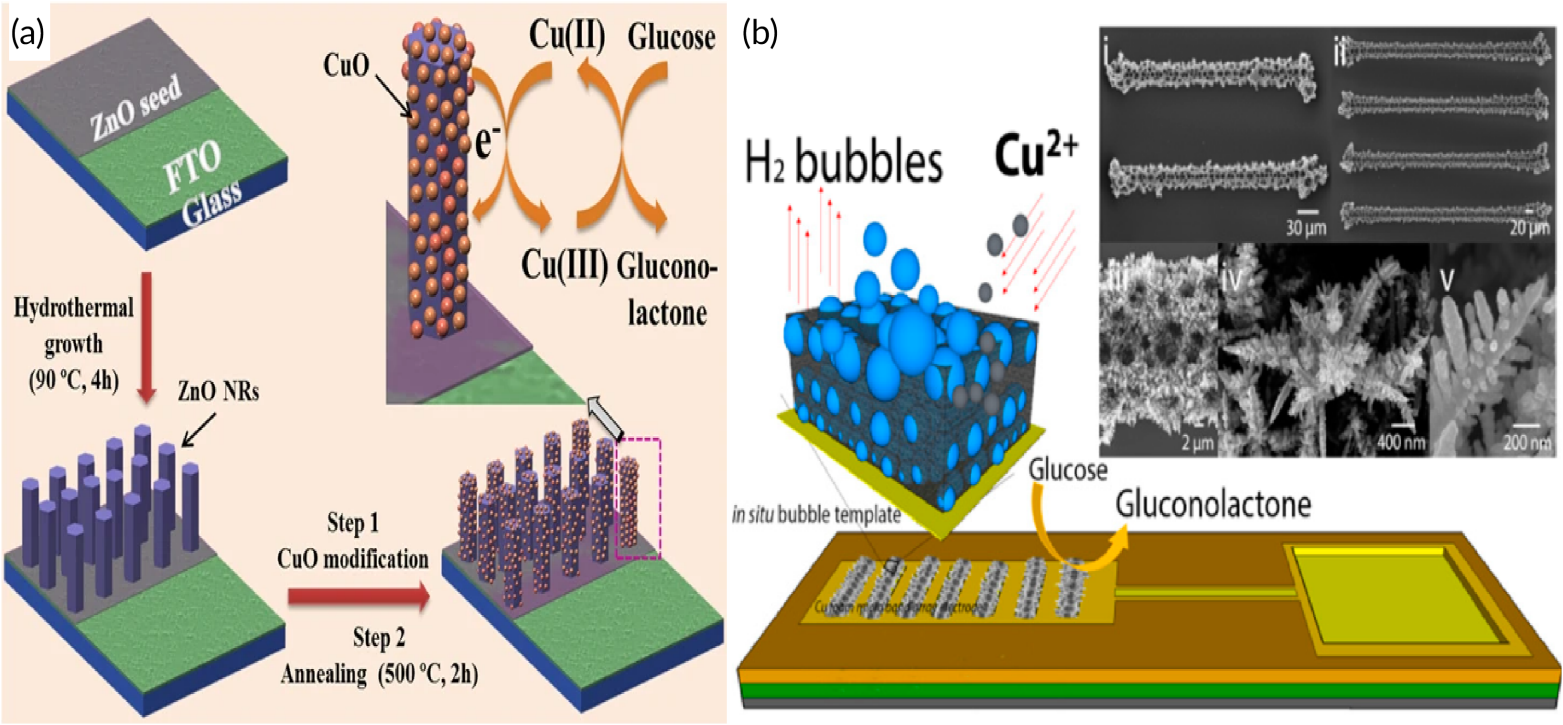
Nonenzymatic glucose (NEGS) with copper**‐**based electrodes. (a) Fabrication and application of NEGS for glucose detection. Adapted with permission from Reference 122, Copyright @ 2017 (Nature). (b) Hydrogen bubble template‐based electrodeposition process of the Cu foam and the SEM images of the resultant Cu Foam electrodeposits. Adapted with permission from Reference 171, Copyright @ 2019 (American Chemical Society)

**FIGURE 9:**
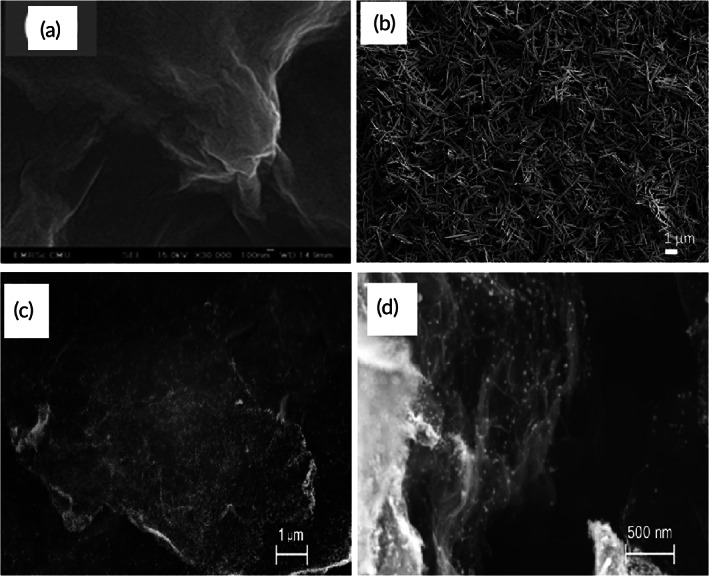
Field emission scanning electron microscopy (FESEM) characterization of copper particles used in nonenzymatic glucose (NEGS). (a) Bare Cu(II)/GO modified SPCE. Adapted with permission from Reference 172, Copyright @ 2021 (Nature). (b) Engineered hierarchical CuO nanoleaves. Adapted with permission from Reference 104, Copyright @ 2021 (IOP Science). (c,d) Copper nanoparticles electrochemically deposited on the PRG sheets. Adapted with permission from Reference 173, Copyright @ 2015 (PLos One)

To reduce the drawbacks incurred by these EGS and the volatile nature of enzymes, the NEGS were introduced (Figure 8). As discussed earlier, these sensors involve direct electrocatalytic oxidation of glucose molecules on their electrodes' surface. Because of their high electrocatalytic activity and advantages like inexpensive availability, nontoxic nature, ability to be quickly processed, and readily stored, copper, copper oxides‐based nanomaterials, and their hybrids have sparked significant interest for FGGS, too.^145^ For example, Zhang et al. developed a nonenzymatic glucose‐sensing platform based on one‐dimensional Cu NWs, both sensitive and selective.^146^ Wang et al. created a sensitive FGGS using CuO flowers and nanorods as the sensing material.^147^ Moreover, significant efforts have been made to combine copper or copper oxides with carbon‐based nanomaterials to enhance their catalytic activity.^148^, ^149^ Luo et al. designed an FGGS based on Cu–graphene nanocomposites, which demonstrated a significantly higher current and a lower negative onset potential for glucose oxidation than Cu NPs.^148^ Field emission SEM (FESEM) analysis of copper particles helps analyze the arrangement of the physical features of the crystalline particles (Figure 9).

Nanozymes have also gained popularity in recent decades. These nanozymes are nanomaterials that possess properties akin to enzymes and have been extensively studied for sensing purposes.^150^ Wei and Wang were the first to equate catalytic NPs with artificial enzymes.^151^ However, like EGS, NEGS face a few setbacks that hinder their practical application at a clinical level. This includes their lowered selectivity because of the lack of a prominent recognition element in the device, and their ability to function correctly under highly alkaline conditions, which means that these sensors will not show their best function within physiological pH. Also, most recent studies have focused on improving the material structure of nanomaterials used in NEGS and less work has been done to enhance targeted and highly sensitive glucose detection. EGS, on the contrary, have shown to give better glucose detection results. Therefore, the scientific focus must steer toward a better understanding of the mechanisms involved in the catalytic processes. Also, further works need to be done to explore other ways to develop nanomaterials that mimic enzymes like in EGS, and possess versatile 3D structures and have better application in the sensing process.

## 4 CONCLUSION AND FUTURE PERSPECTIVE

Recent advances in the fabrication of FGGS have significantly improved. However, the practical application of these devices continues to face significant challenges and hurdles. Efforts are being made to investigate Cu as a competing electrode for FGGS by improving the surface area, shape, and size to volume ratio, enhancing catalytic properties and stability, and detection capability. Reduced stability, shorter shelf life, and enzyme denaturation have limited the application of EGS, focusing researchers toward the tremendously growing field of FGGS. Even though numerous articles have been published demonstrating the efficacy of transition elements as electrocatalytic nanostructures for FGGS advancement, enzyme‐based glucose sensors still outperform the former category for their sensitivity and biocompatibility. To commercially enlist the FGGS, copper‐based characteristics such as low cost, stability, simplicity, and natural abundance can be leveraged to achieve the central goal. When exposed to air, Cu‐based biosensors are easily oxidized, reducing their stability. This can be improved by incorporating other nanomaterials within them, but this complicates FGGS fabrication.

Cu‐based electrodes perform best in alkaline media that operate in a synergistic environment, though the exact mechanism remains unknown. The researchers hope to develop FGGS to detect low glucose levels in blood samples and other bodily fluids. These significant challenges in making Cu‐based glucose sensors stable, reproducible, competitive, and commercially available with EGS are within reach, but the field remains exciting. Cu and its oxides are among the best electrocatalytic nanostructures for manufacturing glucose biosensors, but complete dedication is required to eliminate the shortcomings mentioned above. These efforts should be taken seriously to overcome the difficulties associated with maintaining optimal glucose levels in the blood. These findings contribute to the investigation of future research for the development of advanced versions of Cu‐based FGGS.

## CONFLICT OF INTEREST

The authors declare no potential conflict of interest.

## AUTHOR CONTRIBUTIONS

**Tasbiha Awan:** Data curation (equal); methodology (equal). **Gowhar Naikoo:** Conceptualization (equal); investigation (equal); writing–original draft (equal). **Hiba Salim:** Formal analysis (equal); methodology (equal). **Fareeha Arshed:** Formal analysis (equal); methodology (equal). **Israr Hassan:** Data curation (equal); formal analysis (equal); methodology (equal). **Mona Pedram:** Formal analysis (equal); methodology (equal). **Waqar Ahmed:** Formal analysis (equal); methodology (equal). **Hakkim Faruck:** Data curation (equal); investigation (equal); methodology (equal). **Alaa Aljabali:** Investigation (equal); writing–original draft (equal); writing–review and editing (equal). **Vijay Mishra:** Investigation (equal). **Ángel Serrano‐Aroca:** Formal analysis (equal); investigation (equal); methodology (equal). **Rohit Goyal:** Formal analysis (equal); investigation (equal); methodology (equal). **Poonam Negi:** Investigation (equal). **Martin Birkett:** Investigation (equal); writing–review and editing (equal). **Mohamed Nasef:** Writing–review and editing (equal). **Nitin Charbe:** Writing–review and editing (equal). **Hamid A. Bakshi:** Conceptualization (equal); investigation (equal); project administration (equal); supervision (equal); writing–original draft (equal); writing–review and editing (equal). **Murtaza Tambuwala:** Conceptualization (equal); investigation (equal); project administration (equal); supervision (equal); writing–original draft (equal); writing–review and editing (equal). [Correction added on September 24, 2021 after first online publication: Contribution details of Hamid A. Bakshi has been added.]

### PEER REVIEW

The peer review history for this article is available at https://publons.com/publon/10.1002/btm2.10248.

## Data Availability

Data sharing not applicable to this article as no datasets were generated or analysed during the current study.
